# Saturated Alanine Scanning Mutagenesis of the Pneumococcus Competence Stimulating Peptide Identifies Analogs That Inhibit Genetic Transformation

**DOI:** 10.1371/journal.pone.0044710

**Published:** 2012-09-13

**Authors:** Chaohui Duan, Luchang Zhu, Ying Xu, Gee W. Lau

**Affiliations:** 1 Department of Pathobiology, University of Illinois at Urbana-Champaign, Urbana, Illinois, United States of America; 2 Laboratory of Clinical Immunology, Sun Yat-Sen Memorial Hospital, Sun Yat-Sen University, Guangzhou, Guangdong, People’s Republic of China; University of Iowa Carver College of Medicine, United States of America

## Abstract

Antibiotic resistance is a major challenge to modern medicine. Intraspecies and interspecies dissemination of antibiotic resistance genes among bacteria can occur through horizontal gene transfer. Competence-mediated gene transfer has been reported to contribute to the spread of antibiotic resistance genes in *Streptococcus pneumoniae*. Induction of the competence regulon is mediated by a 17-amino acid peptide pheromone called the competence stimulating peptide (CSP). Thus, synthetic analogs that competitively inhibit CSPs may reduce horizontal gene transfer. We performed saturated alanine scanning mutagenesis and other amino acid substitutions on CSP1 to screen for analogs that disable genetic transformation in *S. pneumoniae*. Substitution of the glutamate residue at the first position created analogs that could competitively inhibit CSP1-mediated competence development in a concentration-dependent manner. Additional substitutions of the negatively-charged glutamate residue with amino acids of different charge, acidity and hydrophobicity, as well as enantiomeric D-glutamate, generated analogs that efficiently outcompeted CSP1, suggesting the importance of negative charge and enantiomericity of the first glutamate residue for the function of CSP1. Collectively, these results indicate that glutamate residue at the first position is important for the ability of CSP1 to induce ComD, but is dispensable for the peptide to bind the receptor. Furthermore, these results demonstrate the potential applicability of competitive CSP analogs to control horizontal transfer of antibiotic resistance genes in *S. pneumoniae*.

## Introduction

*Streptococcus pneumoniae* is a major cause of numerous diseases worldwide, including pneumonia, otitis media, meningitis, bacteremia and sepsis [1]. In recent decades, antibiotic resistant *S. pneumoniae* have been increasingly isolated in clinical settings [2]–[8]. Genetic transformation, which occurs when *S. pneumoniae* enters the competent state, contributes to the transfer and acquisition of antibiotic resistance genes [9]–[11]. The competence regulon is activated when CSP binds to its membrane-associated histidine kinase receptor ComD, which phosphorylates the response regulator ComE [12]–[15]. In turn, ComE activates the transcription of genes in the competence regulon, including the transcription of ComX, a competence specific sigma factor. ComX positively regulates the transcription of genes encoding effectors for DNA uptake and recombination [16]. Interestingly, we and others have shown that the competence regulon is also important for virulence during acute pneumonia and bacteremia models of mouse infection [17]–[22].

DNA sequence analysis predicts the existence of six distinct CSP subtypes, with an overwhelming majority of *S. pneumoniae* strains producing CSP1 or CSP2 [23], [24], corresponding to two variants of ComD, namely ComD1 and ComD2 [25]. Competence in ComD1 strains could be induced more efficiently with the “compatible” CSP1. In contrast, ComD2 strains are more sensitive to induction by the “compatible” CSP2 [26], [27].

Alanine scanning was previously used to identify amino acid residues that are important for the activity of CSP1 [28]. However, the author did not test the ability of these analogs to competitively inhibit CSP1. Most recently, by using amino acid substitution and deletion, we have identified synthetic analogs of CSP1 and CSP2 that competitively inhibit CSPs’ ability to induce the competence regulon, to control pneumococcal pneumonia and to reduce horizontal gene transfer of an antibiotic resistance gene during infection [27]. In this study, we performed a saturated alanine scanning mutagenesis of CSP1, as well as substitution of amino acids based on differing charges, acidity, hydrophobicity and enantiomericity, and examined the ability of these analogs to inhibit the development of competence.

## Results

### Saturated Alanine Mutagenesis Screen Identifies CSP1 Analogs That are Unable to Induce *comX* Expression and Genetic Transformation

**Figure 1:**
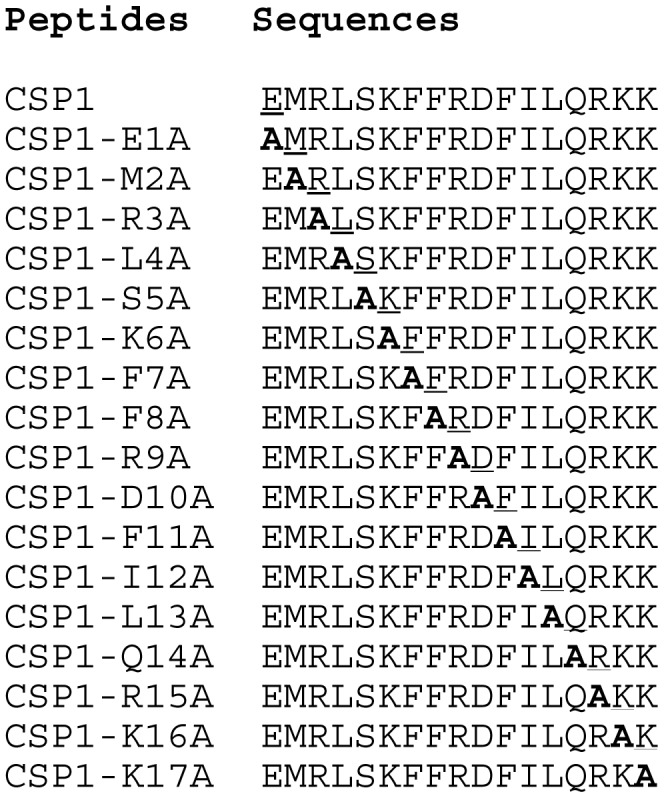
Amino acid sequences of CSP1 and its analogs. Analogs were synthesized based on the sequence of CSP1 by alanine substitutions.

**Figure 2:**
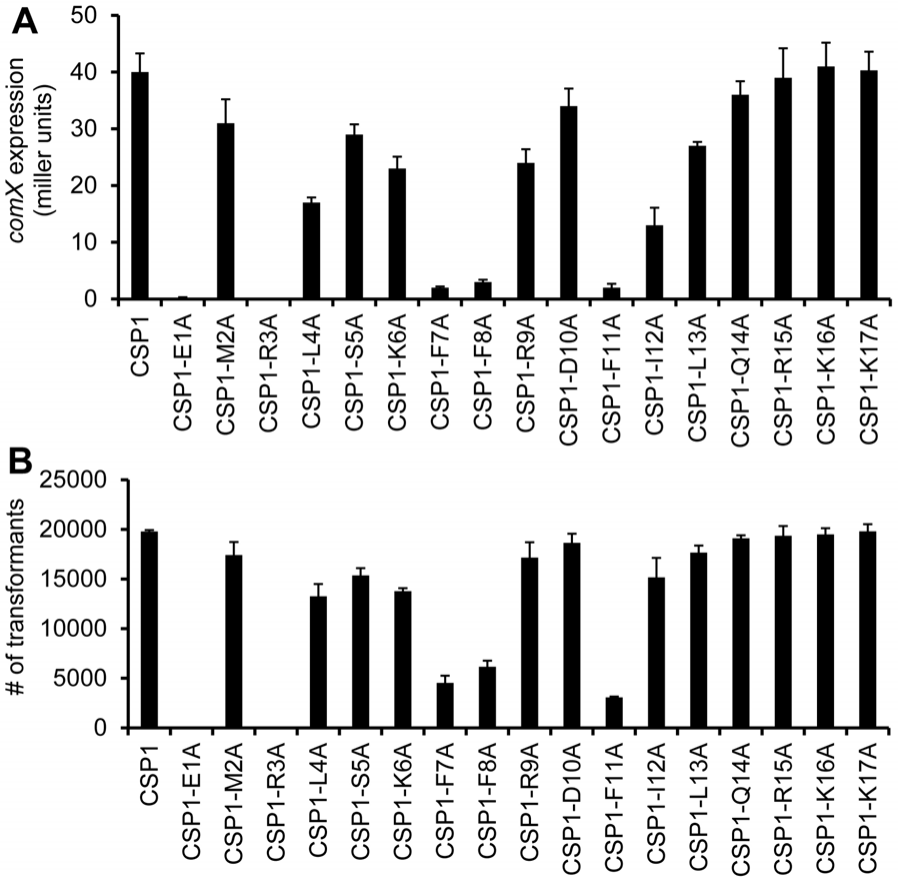
Analyses of the ability of CSP1 analogs to induce *comX* expression and genetic transformation. Pneumococcal cells were incubated with CSP1 or with each analog at a final concentration of 100 ng/ml. The ability of each peptide to induce the competence regulon was measured by induction of the *comX* expression using the ß-galactosidase assays in D39pcomX::*lacZ* cells (A) or by genetic transformation using the *rpsL* gene in D39 cells (B). Transformants were selected on THB agar containing 100 µg/ml streptomycin. Experiments were performed in triplicates and repeated three times. The means ± SD of one typical experiment are shown.

We performed a saturated alanine scanning mutagenesis on CSP1 to identify additional synthetic analogs that would outcompete CSP1 and attenuate competence development and genetic transformation (Fig. 1). The ability of CSP1 and its analogs to induce competence in the wild-type *S. pneumoniae* strain D39 was compared by monitoring the: (i) promoter activity of *comX*, (ii) transformation frequency of *rpsL* gene that confers resistance to streptomycin [29]. Induction of ComX indicates that *S. pneumoniae* cells have entered the competent state for genetic transformation. Activation of the *comX* gene promoter was monitored by assaying ß-galactosidase activity in pneumococcal strain D39pcomX::lacZ [27]. The ability of several CSP1 analogs to activate the expression of *comX*, including CSP1-E1A, CSP1-R3A, CSP1-F7A, CSP1-F8A and CSP1-F11A, were attenuated by 95.5, 100, 95, 92.5, 95%, respectively (Fig. 2A). Because ComX regulates DNA uptake and transformation, we examined the ability of all analogs to induce genetic transformation. Consistent with their reduced ability to induce the expression of *comX*, CSP1-E1A, CSP1-R3A, CSP1-F7A, CSP1-F8A and CSP1-F11A were attenuated in their ability to induce genetic transformation, by 99.9, 100, 77.1, 68.9 and 84.5%, respectively (Fig. 2B).

### CSP1-E1A is An Analog that Competitively Inhibits CSP1

**Figure 3:**
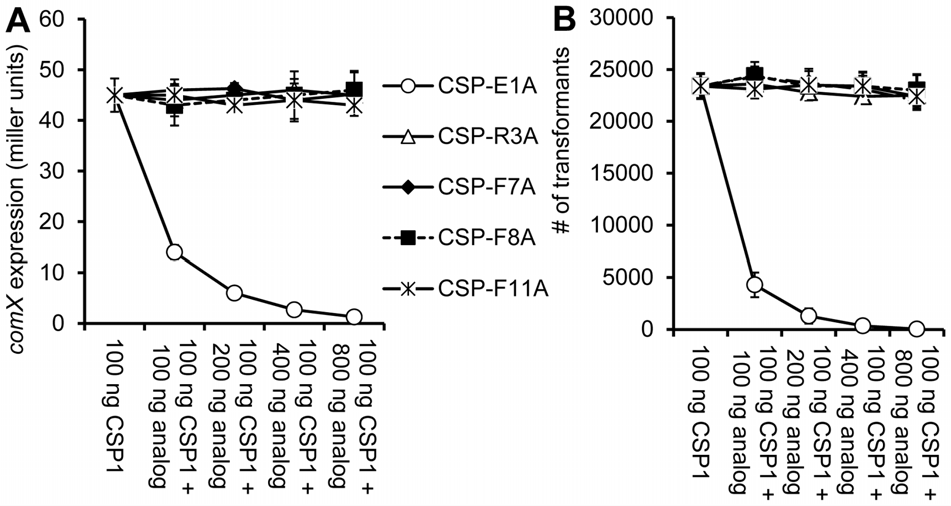
CSP1-E1A is uniquely able to inhibit the expression of *comX* and genetic transformation. (A) CSP-E1A competitively inhibits CSP1-mediated induction of *comX* in a dose dependent manner. D39pcomX::lacZ cells were exposed to 100 ng/ml of CSP1 alone or simultaneously with increasing concentrations of each CSP1 analog. The activity of the *comX* gene promoter was measured by β-galactosidase activity. (B) CSP-E1A competitively inhibits CSP1-mediated genetic transformation. Genetic transformation was performed using 30 µg/ml of the D39 genomic DNA containing the *rpsL* gene. Transformants were selected on THB agar containing 100 µg/ml streptomycin. Experiments were performed in triplicates and repeated three times. The means ± SD of one typical experiment are shown.

Because of their inability to induce *comX* expression and genetic transformation, we next determined whether these five analogs could competitively inhibit wild type CSP1. Analogs CSP1-R3A, CSP1-F7A, CSP1-F8A and CSP1-F11A failed to inhibit CSP1-induced *comX* expression (Fig. 3A), suggesting that these analogs were incapable of binding to ComD1 receptor. In contrast, CSP1-E1A, with glutamate residue at the first position substituted with alanine, competitively inhibited CSP1 in a concentration-dependent manner. The inductive activity of 100 ng CSP1 on *comX* expression was inhibited by 69, 86.7, 94 and 97% in the presence of 100, 200, 400 or 800 ng of CSP1-E1A, respectively (Fig. 3A). Similarly, CSP1-mediated genetic transformation was inhibited by 81.6, 94.4, 98.5 and 99.8% in the presence of indicated concentrations of CSP1-E1A, respectively (Fig. 3B). These results suggest that among the five CSP1 analogs with defective ability to activate the competence regulon, only CSP1-E1A is uniquely capable of competitively inhibiting CSP1.

### Negative Charge and Enantiomericity of the First Glutamate Residue are Important for the Activity of CSP1

**Figure 4:**
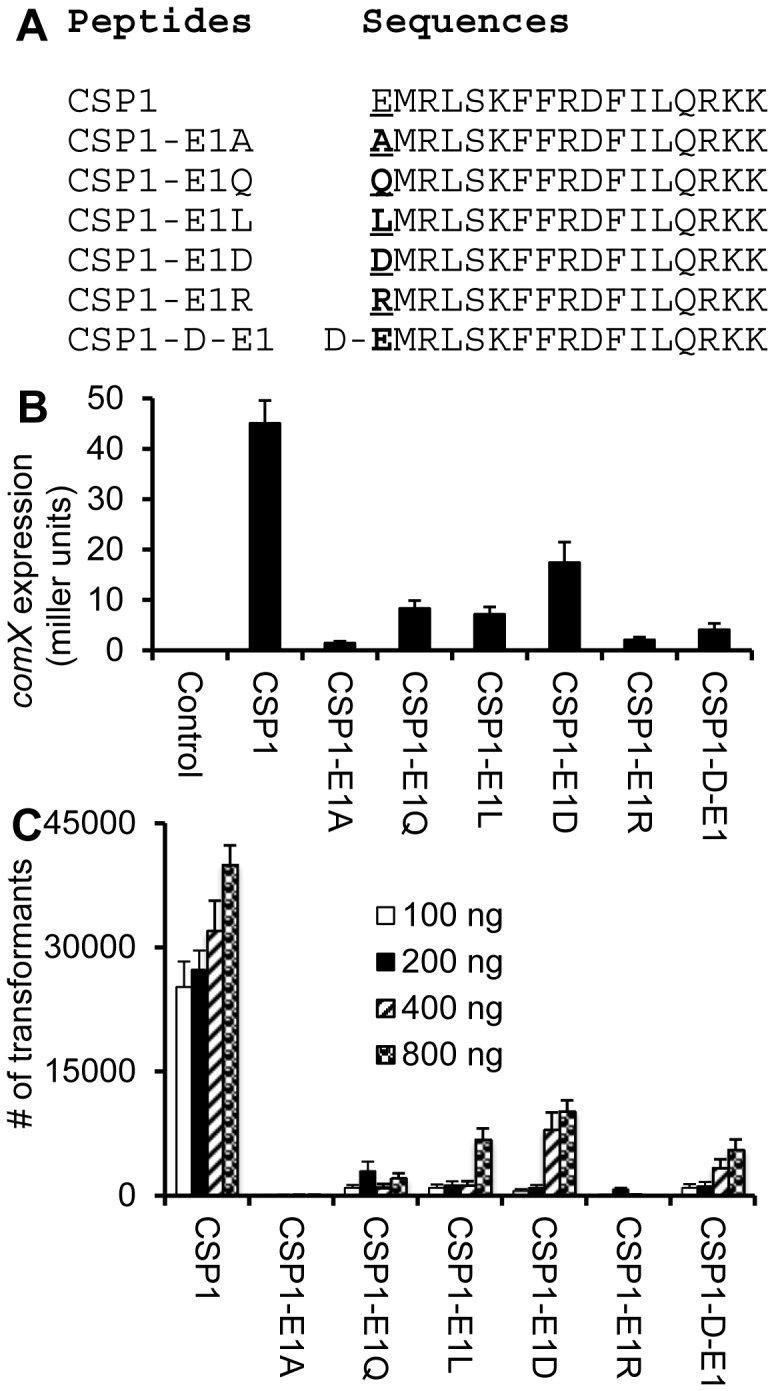
The negative charge and enantiomericity of glutamate residue in the first position is important for the activity of CSP1. (A) Amino acid substitutions of the first glutamate residue in CSP1. (B) Induction of *comX* expression by CSP1 analogs. D39pcomX::*lacZ* were incubated with CSP1 or with each glutamate substituted analog at a final concentration of 100 ng/ml. The ability of each peptide to induce the competence regulon was measured by induction of the *comX* by ß-galactosidase assays. (C) Genetic transformation of D39 cells in the presence of indicated concentrations of CSP1 and its analogs using the *rpsL* gene. Transformants were selected on THB agar containing 100 µg/ml streptomycin. Experiments in B and C were performed in triplicates and repeated three times. The means ± SD of one typical experiment are shown.

Glutamate is polar and negatively charged. Substitution of glutamate with alanine (nonpolar, neutral) at the first position generates a strong competitive inhibitor of CSP1 (Figure 3). This observation suggests that the negative charge of glutamate is important for the activity of CSP1, but dispensable for the binding to ComD1. To determine the importance of negative charge on glutamate to the activity of CSP1, we examined five additional synthetic analogs (Fig. 4A), by substituting the glutamate with glutamine (CSP1-E1Q, polar, neutral), leucine (CSP1-E1L, non-polar, neutral), aspartic acid (CSP1-E1D, polar, negatively-charged), and arginine (CSP1-E1R, polar, positively-charged), and an enantiomeric analog using the D-glutamate (CSP1-D-E1, polar, negatively-charged) (Fig. 4A), for their ability to competitively inhibit CSP1. All five new analogs were attenuated in their ability to induce the expression of *comX* (Fig. 4B). Importantly, the analog CSP1-E1D, in which the glutamate residue is substituted with another polar and negatively-charged amino acid aspartate, retained 35.6% of the wild-type CSP1 activity to induce the expression of *comX* (Fig. 4A), confirming that negative charge of the glutamate residue is important for the activity of CSP1. Concurring with this hypothesis, we showed that CSP1-E1R (polar, positive-charged arginine) was most attenuated, with the levels of *comX* induction and genetic transformation reduced to 4.6% and 0% (800 ng/ml), respectively (Fig. 4A–B). In addition, CSP1-E1L and CSP1-E1Q were also able to induce *comX* expression and genetic transformation, but to lesser degrees than CSP1-E1D. Finally, substitution of L-glutamate in the first position of CSP1 with its enantiomeric isomer D-glutamate also impaired its ability to induce *comX* expression and genetic transformation (Fig. 4A–B). Collectively, these results suggest that negative charge and structural conformation in the N-terminus of CSP1 are important for its activity.

### Differential Amino Acid Substitutions of Glutamate at the First Position Generate New Inhibitors of CSP1

**Figure 5:**
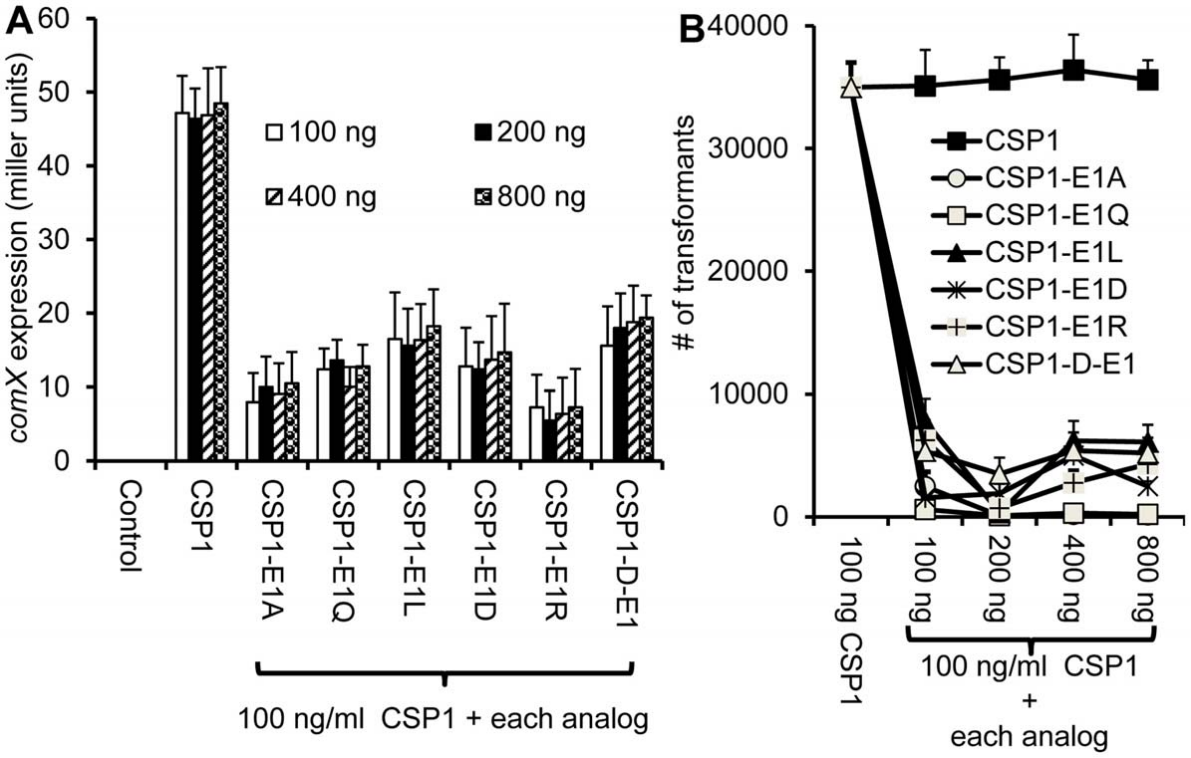
Dose dependent inhibition of *comX* expression and genetic transformation by CSP1 analogs with substitution at glutamate residue in the first position. (A) CSP1 analogs with substitution at the first glutamate acid competitively inhibit CSP1-mediated induction of *comX*. D39pcomX::lacZ cells were exposed to 100 ng/ml of CSP1 alone or simultaneously with increasing concentrations of CSP1 analogs. The activity of the *comX* gene promoter was measured by β-galactosidase activity. Experiments were performed in triplicates and repeated three times. The means ± SD of one typical experiment are shown. (B) CSP1 analogs with substitution at the first glutamate acid competitively inhibit the ability of CSP1 to induce genetic transformation. Genetic transformation was performed using the *rpsL* gene. Transformants were selected on THB agar containing 100 µg/ml streptomycin. Experiments were performed in triplicates and repeated three times. The means ± SD of one typical experiment are shown.

**Figure 6:**
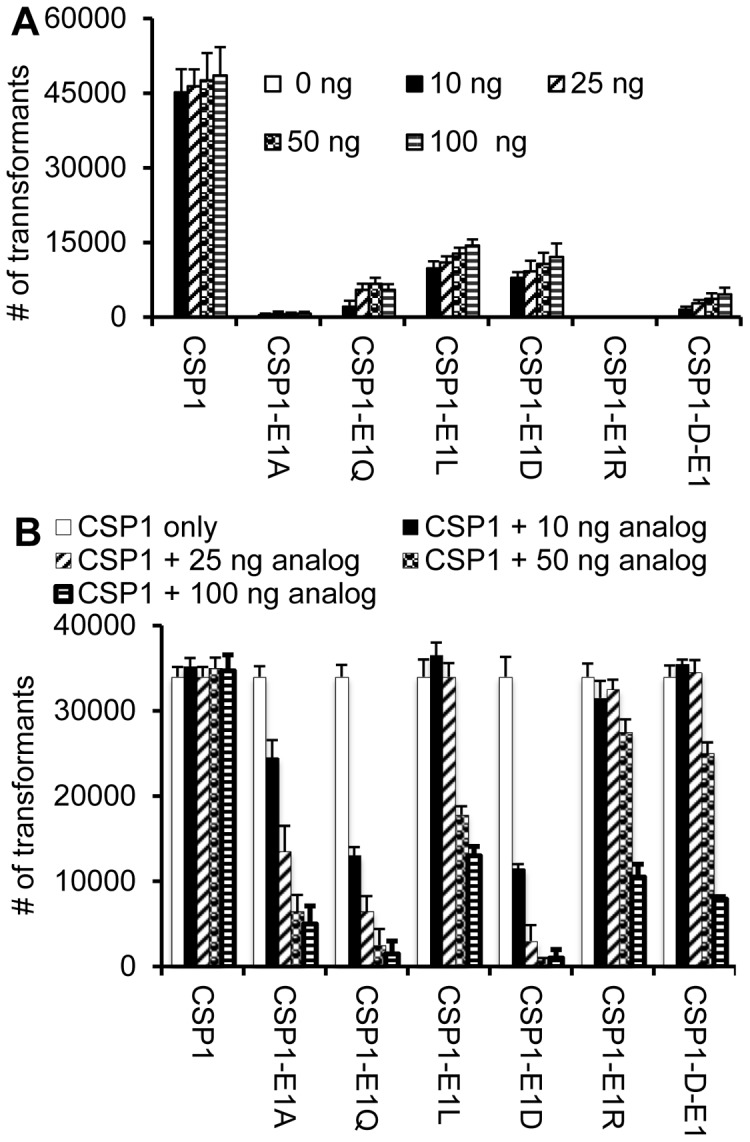
Competitive inhibition of CSP1 by low concentrations of analogs with substitutions of glutamate residue in the first position. (A) Induction of genetic transformation by each analog using the genomic DNA containing the *rpsL* gene. Transformants were selected on THB agar containing 100 µg/ml streptomycin. Experiments were performed in triplicates and repeated three times. The means ± SD of one typical experiment are shown. (B) Competitive inhibition of genetic transformation by low concentrations of CSP1 analogs. Genetic transformation was performed using the genomic DNA containing the *rpsL* gene. Transformants were selected on THB agar containing 100 µg/ml streptomycin. Experiments were performed in triplicates and repeated three times. The means ± SD of one typical experiment are shown.

Next, we examined whether these CSP1 analogs with substitution at the glutamate residue could competitively inhibit the induction of *comX* and genetic transformation. All analogs inhibited *comX* induction and ComX-dependent genetic transformation to similar levels (Fig. 5A–B). The lack of distinction between the ability of CSP1-E1A and CSP1-E1R versus CSP1-E1D was surprising, probably caused by high amounts of analogs (100 ng/ml) that were used for these assays. To overcome the aforementioned difficulty, we examined CSP1 inhibition using lower concentrations of these analogs (Fig. 6). Analogs CSP1-E1R, CSP1-D-E1 and CSP1-E1Q had low ability to induce genetic transformation (Fig. 6A). Interestingly, at low concentrations, CSP1-E1Q and CSP1-E1D were found to be most effective at inhibiting CSP1-mediated transformation (Fig. 6B). Surprisingly, CSP1-E1R, which had the lowest ability to induce *comX* and genetic transformation (Figs. 6A), was least effective in inhibiting genetic transformation at low peptide concentrations (Fig. 6B). Conversely, CSP1-E1D, which retained one of the highest activities to induce *comX* expression and genetic transformation (Fig. 6A), was the most effective competitive inhibitor at low peptide concentrations (Fig. 6B). Finally, CSP1-E1Q, which was highly attenuated in its ability to induce *comX* expression and genetic transformation (Fig. 6A), was as effective as CSP1-E1D at competitively inhibiting CSP1 at low peptide concentrations (Fig. 6B).

## Discussion

The competence regulon regulates genetic transformation [11], [30] and virulence in *S. pneumoniae* [17]–[22]. In this study, we used saturated alanine scanning to identify synthetic analogs of CSP1 that could competitively inhibit genetic transformation of an antibiotic resistance gene. Substitutions of the 1^st^ (CSP1-E1A), 3^rd^ (CSP1-R3A), 7^th^ (CSP1-F7A), 8^th^ (CSP1-F8A) and 11^th^ (CSP1-F11A) amino acid residues with alanine generate synthetic analogs with impaired ability to induce the competence regulon. However, only substitutions of the glutamate residue at the first position generate analogs that competitively inhibit CSP1. These results suggest that the glutamate residue is important for the ability of CSP1 to induce ComD, but is dispensable for binding to the histidine kinase receptor.

The chemical properties of individual amino acid residues, including charges, acidity, polarity and hydrophobicity may influence the activity of a peptide. Glutamate is negatively charged. The importance of negative charge is demonstrated by the analog CSP1-E1D, which has the glutamate in the first position substituted with negatively charged aspartate, preserves significant amounts of ability to induce competence regulon. This suggests that CSP1-E1D retains significant amount of binding to ComD1, and is thus, paradoxically, more efficient in competitively displacing the binding of CSP1 to ComD1. This argument is supported by the observation that CSP1-E1R, which the glutamate has been substituted with positively-charged arginine (CSP1-E1R), is least effective in inhibiting CSP1. Another highly effective inhibitory analog is CSP1-E1Q. Glutamine is neutrally charged, yet structurally most similar to glutamate. Structural similarity and lesser charge difference between glutamate-glutamine versus glutamate-arginine may have contributed to the different competitiveness between CSP1-E1Q versus CSP1-E1R. Because of its low ability to induce competence and genetic transformation, yet strong ability to competitively inhibit CSP1, we predict that CSP1-E1Q is the best inhibitor analog. Finally, the L-glutamate conformation is also important because enantiomeric substitution with D-glutamate severely reduces the activity of CSP1.

The use of inhibitory analogs of CSP1 may have an application in reducing horizontal transfer of antibiotic resistance genes. For example, competence in genetic transformation is induced by CSP1 and other peptide pheromones in streptococcal species by two major systems [13]. These systems differ in their methods of inducing the expression of ComX. Streptococcal species in Anginosus and Mitis groups use the ComCDE system to induce *comX* expression. Thus, CSP analogs that inhibit ComD to suppress competence may be applicable in these streptococcal species. The second system, ComRS, is utilized by streptococcal species in Pyogenic, Salvarius and Bovis groups [31]. In this system, the precursor of the competence peptide ComS is cleaved by an extracellular protease, and the processed peptide re-enters the cytoplasm through peptide permease to activate the cytoplasmic transcriptional regulator ComR to initiate the expression of *comX*. It will be interesting to explore if analogs of ComS could inhibit competence in the relevant streptococcal species.

In recent decades, incidence of *S. pneumoniae* resistance to β-lactams, macrolides, and other classes of antibiotics has escalated dramatically [2]–[8]. While conjugative elements appear to be more important for the spread of antibiotic resistance genes [4], [32]–[34], horizontal gene transfer of penicillin binding protein genes has occurred in clinical isolates of *S. pneumoniae* [10]. Importantly, it has been shown that under *in vitro* conditions, antibiotic stress stimulates horizontal gene transfer in bacteria, including *S. pneumoniae* [35]–[37]. For example, β-lactams induce the SOS response and horizontal transfer of virulence factors in *Staphylococcus aureus* [36]. Similarly, DNA damaging antibiotics trigger genetic exchange in *Helicobacter pylori* [35]. In this case, a coupling agent that inhibits horizontal gene transfer reduces the risk of generating antibiotic resistant pathogens during antibiotic treatment. In *S. pneumoniae*, antibiotic stress has been reported to induce the competence regulon and increase genetic transformation [37]. These authors also reported that induction of competence by antibiotic stress does not occur in a *comA* mutant that lacks the ABC transporter needed to export CSP, suggesting that this process is CSP-dependent. Collectively, these studies suggest that under certain clinical conditions, *S. pneumoniae* actively exploits the opportunity of antibiotic stress to acquire exogenous genes. Thus, antibiotic resistance mediated by the competence regulon may be under reported and worth more detailed investigations.

In conclusion, our data show that CSP analogs can effectively attenuate genetic transformation in *S. pneumoniae*. This strategy may be applicable to reduce the incidence of horizontal gene transfer and acquisition of antibiotic resistance genes. In addition, because competence systems in some streptococcal species, including *S. mitis,* are very similar to that of *S. pneumoniae*, there is a possibility that the former could be transformed by pneumococcal DNA [38], [39]. *S. mitis* is generally considered an avirulent species but has the potential to acquire virulence determinants from *S. pneumoniae* and transform itself into a pathogen. In this scenario, CSP1 analogs may be applicable to reduce the emergence of the new pathogen.

## Materials and Methods

### Bacterial Strains and Growth Conditions

*S. pneumoniae* strain D39 [40] was a generous gift from Dr. David Briles (University of Alabama-Birmingham). Strain D39pcomX::lacZ was generated by transforming D39 with the genomic DNA from CPM3 that harbored an insertion of the *lacZ* gene under the control of the *comX* promoter [27]. Aliquots of bacteria were stored at −80°C in Todd Hewitt broth (THB) containing 25% glycerol. For routine experiments, bacteria from frozen stocks were streaked onto THB agar containing 5% defibrinated horse blood and incubated for 12–24 hr at 37°C with 5% CO_2_. Fresh colonies were transferred to THB and cultured to desired density as measured by a spectrophotometer at OD 600 _nm_.

### Synthetic CSPs

Both CSP1 and its analogs (≥95% purity) were synthesized by Elim Biopharm (Hayward, CA).

### Activation Assay of ***comX***

The ability of synthetic CSP-1 and its analogs to activate the expression of *comX* gene was compared in D39pcomX::lacZ cells grown in THB (pH 6.8) until OD 600 _nm_ of 0.1, washed and resuspended in THB (pH 8.3). CSP1 and their analogs were added at indicated concentrations to the culture and incubated at 37°C for 30 min. ß-galactosidase activity in pneumococcal cells was measured according to previously published protocols [26], and expressed as Miller units.

### ***S. pneumoniae*** Transformation Assay

Genetic transformation experiments were performed as we had previously described [18], [26]. Briefly, *S. pneumoniae* cells were grown to early log phase (OD 600 _nm_ ∼ 0.1) in THB (pH 6.8), washed and resuspended in THB (pH 8.3) containing 30 µg/ml of D39 genomic DNA carrying a copy of the streptomycin resistance *rpsL* gene originated from strain CP1296 [41]. Transformation experiments were performed in the present CSP1 or its analogs. The transformation mix was incubated at 37°C with 5% CO_2_ for 1 hr. Transformants were selected on THB agar supplemented with 100 µg/ml streptomycin.
